# Health systems developments and predictors of bystander CPR in Ireland

**DOI:** 10.1016/j.resplu.2024.100671

**Published:** 2024-05-31

**Authors:** Tomás Barry, Alice Kasemiire, Martin Quinn, Conor Deasy, Gerard Bury, Siobhan Masterson, Ricardo Segurado, Andrew W Murphy, Out-of-Hospital Cardiac Arrest Registry Steering Group

**Affiliations:** a School of Medicine, University College Dublin, Ireland; b Duke-NUS Medical School, Singapore; c Statistician, UCD Centre for Support and Training in Analysis and Research, School of Public Health, Physiotherapy and Sports Science, University College Dublin, Dublin, Ireland; d Out-of-Hospital Cardiac Arrest Register, National Ambulance Service, Ireland; e Professor of Emergency Medicine, School of Medicine, University College Cork, Cork, Ireland; f Emeritus Professor of General Practice, University College Dublin, Ireland; g General Manager for Clinical Strategy and Evaluation National Ambulance Service, Ireland; h UCD Centre for Support and Training in Analysis and Research, School of Public Health, Physiotherapy and Sports Science, University College Dublin, Dublin, Ireland; i Foundation Professor of General Practice, Discipline of General Practice & HRB Primary Care Clinical Trials Network Ireland, University of Galway, Galway, Ireland

**Keywords:** Resuscitation, Out-of-Hospital Cardiac Arrest, Cardiopulmonary Resuscitation, Bystander CPR, Registry Data, Statistical Models, Public Health

## Abstract

**Aims:**

To explore predictors of bystander CPR (i.e. any CPR performed prior to EMS arrival) in Ireland over the period 2012–2020. To examine the relationship between bystander CPR and key health system developments during this period.

**Methods:**

National level out-of-hospital cardiac arrest (OHCA) registry data relating to unwitnessed, and bystander witnessed OHCA were interrogated. Logistic regression models were built, then refined by fitting predictors, performing stepwise variable selection and by adding pairwise interactions that improved fit. Missing data sensitivity analyses were conducted using multiple imputation.

**Results:**

The data included 18,177 OHCA resuscitation attempts of whom 77% had bystander CPR. The final model included ten variables. Four variables (aetiology, incident location, time of day, and who witnessed collapse) were involved in interactions. The COVID-19 period was associated with reduced adjusted odds of bystander CPR (OR 0.77, 95% CI 0.65, 0.92), as were increasing age in years (OR 0.992, 95% CI 0.989, 0.994) and urban location (OR 0.52, 95% CI 0.47, 0.57). Increasing year over time (OR 1.23, 95% CI 1.16, 1.29), and an increased call response interval in minutes (OR 1.017, 95% CI 1.012, 1.022) were associated with increased adjusted odds of bystander CPR.

**Conclusions:**

Bystander CPR increased over the study period, and it is likely that health system developments contributed to the yearly increases observed. However, COVID-19 appeared to disrupt this positive trend. Urban OHCA location was associated with markedly decreased odds of bystander CPR compared to rural location. Given its importance bystander CPR in urban areas should be an immediate target for intervention.

## Introduction

Performing chest compressions is the only widely available means to provide vital organ perfusion during cardiac arrest.^1^ Early chest compressions by bystanders following out-of-hospital cardiac arrest (OHCA) i.e. bystander cardiopulmonary resuscitation (CPR) is essential to slow the rate of deterioration of the brain and heart and to buy time to enable defibrillation.^2^ When bystander CPR is performed following OHCA, this has been associated with increased survival and decreased brain damage.^3^ Ireland’s population is more than 5.1 million people.^4^ There are now approximately 3,000 OHCA resuscitation attempts in this population each year, 80% involve bystander CPR.^5^ Telephone or dispatch assisted CPR provides a further opportunity to improve survival and good neurological outcomes in circumstances where bystanders have not already initiated CPR at the time of the call to emergency medical services (EMS).^6^ Telephone assisted CPR has been established as a standard of care in Ireland.^7^

**Fig. 1:**
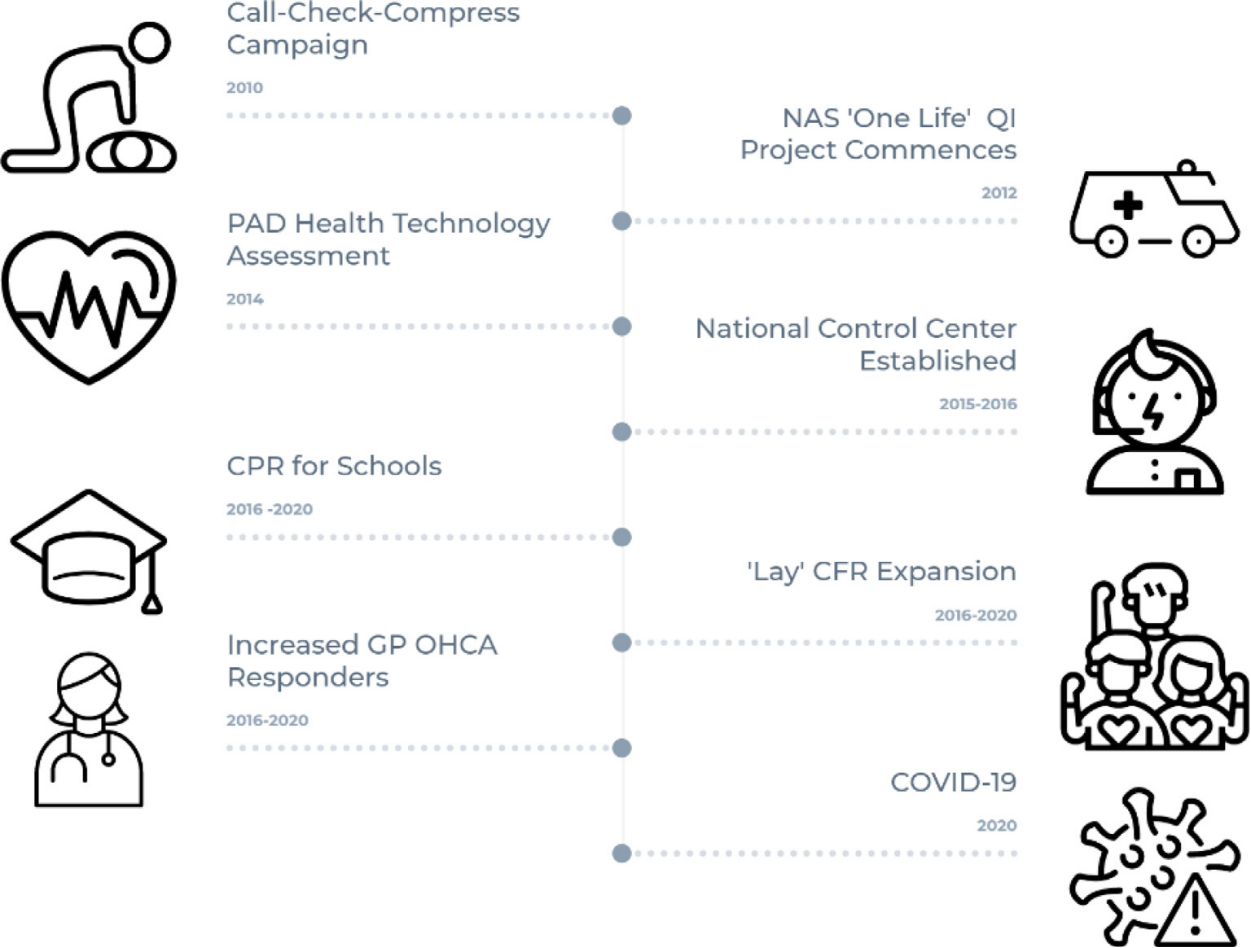
Resuscitation for out-of- hospital cardiac arrest in Ireland 2012–2020: Summary of health system temporal developments. (From − Barry T, Kasemiire A, Quinn M et al. Outcomes of out-of-hospital cardiac arrest in Ireland 2012–2020: Protocol for an observational study [version 2; peer review: 3 approved, 1 approved with reservations]. HRB Open Res 2023, 6:17. (https://doi.org/10.12688/hrbopenres.13699.2)). CFR: Community first responder, CPR: Cardio pulmonary resuscitation, GP: General Practitioner, NAS: National Ambulance Service, OHCA: Out-of-hospital cardiac arrest, PAD: Public access defibrillation,

In recent years the Irish health system has undergone several developments that are relevant to bystander CPR and to wider OHCA care. These developments include public education, training and first responder recruitment campaigns as well as EMS quality improvement initiatives. These are illustrated in Fig. 1 and have previously been described in detail.^8^ Notably, 2015 and 2016 saw a major reconfiguration of National Ambulance Service (NAS) EMS control with multiple independent regional control centres amalgamated into a single entity known as the ‘National Emergency Operations Centre’ (NEOC).^8^ While Dublin Fire Brigade continues to provide the EMS control for the greater Dublin area, this centralisation process for NAS represented a key step in embedding telephone CPR at national level. The year 2020 was notable as the first year of the COVID-19 pandemic, with the initial wave in Ireland between February and July 2020.^8^ As in other jurisdictions, widespread public health and social distancing measures were implemented to limit the spread of infection.^9^ At international level, COVID-19 has been associated with disruption of OHCA care processes and worse survival outcomes.^10^, ^11^ During the COVID-19 pandemic international guidelines continued to recommend that OHCA bystanders provide chest compressions.^12^

Ireland has collected national level data concerning OHCA resuscitation since 2012 via an OHCA registry.^13^ This has facilitated research that contributed to international comparisons of key outcomes including survival and bystander CPR.^14^, ^15^, ^16^ At national level the data has contributed to analysis of public access defibrillation, geographical disparities, first responders and outcomes in key age cohorts.^17^, ^18^, ^19^, ^20^, ^21^, ^22^, ^23^ Prior to this current research, no temporal analyses had been conducted to assess the impact of national level interventions and developments on key OHCA outcomes in Ireland over time. An initial phase of this project has already focused on survival to hospital discharge.^24^ This current element now examines bystander CPR over time in Ireland. The aims of this study were to interrogate Irish OHCA data from the period 2012 to 2020, model predictors of bystander CPR, and explore the impact of relevant health system temporal developments.

## Methods

### OHCA care in Ireland

The National Ambulance Service (NAS) provide statutory OHCA care in Ireland, which in the capital is also delivered by Dublin Fire Brigade.^25^ In tandem with these emergency medical services (EMS), a well-developed national voluntary network of community first responders (CFRs) aims to provide CPR and defibrillation before EMS arrival.^25^ Professional EMS OHCA care is delivered by paramedics and advanced paramedics supported by statutory national guidelines.^26^ CFRs are dispatched by EMS control using SMS alert technology.^25^ They are trained in CPR and are equipped with automatic external defibrillators (AEDs). During the COVID pandemic alerting systems for CFRs were paused owing to safety concerns. In other European countries some CFR schemes were deactivated however others continued to operate although a majority reported decreased activity levels.^27^

### The Irish Out-of-Hospital Cardiac Arrest Register

The Irish Out-of-Hospital Cardiac Arrest Register (OHCAR) achieved national coverage in 2012.^13^ OHCAR employs the Utstein standardised international approach to data collection.^28^ Its primary data sources are EMS dispatch and patient care records while data describing survival to hospital discharge and neurological outcomes are provided by receiving hospitals.^13^ In Ireland EMS patient care records specifically record whether CPR was commenced by an EMS practitioner or whether it was commenced prior to EMS arrival (bystander CPR).

### Study population

The population for this study were patients of all ages who had suffered un-witnessed, or bystander witnessed OHCA during the time period 2012–2020 and who had an EMS resuscitation attempt.

### Research protocol development and variable selection

Prior to data analysis a detailed research protocol was developed and published.^8^ A number of OHCAR variables were identified to be of potential relevance to bystander CPR (defined in this study as any CPR performed prior to EMS arrival). The study team hypothesised that two key temporal developments were likely to be associated with a significant change in bystander CPR. In the first instance, the study team hypothesised that the centralisation of EMS control in 2015 and 2016 with a transition from multiple regional control centres to the single national control centre (NEOC) and enhanced telephone CPR, would be in turn associated with increased bystander CPR.^8^ In addition, given COVID-19′s disruptive effect on OHCA care process internationally, we hypothesised that the COVID-19 period would be associated with decreased bystander CPR in Ireland.^8^ All analyses were based on the variables shown in supplementary Table 1. Variables 1–11 were obtained from OHCAR. Variables 12, 13 and 14 were created using the ‘year’ variable and represent key component time periods that were considered potentially significant. Variable 10 was based on the Irish Central Statistics Office classification of urban or rural. OHCAR variables with multiple associated categories were collapsed to avoid decreased statistical power from analysis of an excessive number of potentially sparse categories. The ‘year’ variable was treated as a continuous variable to conserve degrees of freedom and statistical power.

### Statistical model building

Following the approach set out in our published research protocol^8^ a series of logistic models were built to explore bystander CPR. The effect of each individual variable in the models was summarised using odds ratios and 95% confidence intervals. Initial univariate logistic regression analysis was conducted for each predictor variable. A full multiple logistic model was then fitted incorporating all predictor variables. During the logistic model building process higher order terms (polynomial terms) were added to the model to explore if the relationship between the outcome and the continuous predictors was non-linear. A refined model was then built using stepwise model selection (STEPAIC function in R). During this, several models were built from all possible combinations of predictors by sequentially adding and dropping predictors and ultimately selecting the model with the lowest AIC (Akaike Information Criterion). In the final element of model building, the stepwise model was further improved by adding pairwise interaction variables and retaining interactions which improved model fit. Each model (initial, stepwise, with interactions) was then evaluated by considering AIC, model deviance and the result of the Hosmer-Lemeshow Goodness of Fit (GOF) test to ensure that the overall best performing model was identified. The predictive ability of the final, best fitting model was then evaluated using 10-fold cross-validation.

### Missing data & sensitivity analysis

The proportion of missing data for each variable were documented and evaluated. Sensitivity analyses were conducted using multiple imputation via the mice package in R. This involved multivariate imputation by chained equations using ten imputed data sets and methods appropriate for each variable (binomial or polynomial logistic, or linear regressions). Finally, results from complete case analysis and multiple imputation were compared.

### Ethical approval

The National University of Ireland Galway, Research Ethics Committee provided ethical approval for this study in advance of data processing and analysis (Reference 2020.01.012; Amendment 2106).

## Results

### Missing data

The data contained a total of 18,177 observations. There were some missing data in the outcome variable ‘bystander CPR’ and in predictor variables. In all 1587 observations contained some missing data. Table 1a, Table 1b provide an overview of the proportion of missing data for the outcome variable and all predictor variables across the dataset. The outcome variable relating to bystander CPR was missing in 1.7% of observations. Across the predictor variables the proportion of missing data ranged from 0.0% to 3.5% (urban or rural location).

**Table 1a:** *Bystander CPR in Ireland* in Ireland 2012–2020: Summary of categorical variables.

Variable	Missing		Categories			Outcome Available	Bystander CPR	
	*n*	%		*n*	%	*n*	*n*	%
Chest Compressions Started By	304	1.7	EMS initiated CPR	4106	23.0%			
			Bystander CPR	13,767	77.0%			
			Total	17,873	100.0%			
Aetiology	5	0.0	Presumed Other	2548	14.0%	2486	1815	73.0%
			Presumed Cardiac	15,624	86.0%	15,382	11,947	77.7%
			Total	18,172	100.0%			
Gender	24	0.1	Female	5887	32.4%	5780	4353	75.3%
			Male	12,266	67.6%	12,073	9400	77.9%
			Total	18,153	100.0%			
Incident location	51	0.3	Other Location	5394	29.8%	5321	4470	84.0%
			Home Location	12,732	70.2%	12,501	9257	74.1%
			Total	18,126	100.0%			
Season	0	0.0	Summer	8582	47.2%	8426	6502	77.2%
			Winter	9595	52.8%	9447	7265	76.9%
			Total	18,177	100.0%			
Time of Day	27	0.1	Night	3903	21.5%	3824	2828	74.0%
			Evening	6524	35.9%	6418	5018	78.2%
			Morning	7723	42.6%	7609	5907	77.6%
			Total	18,150	100.0%			
Who Witnessed Collapse	0	0.0	Not Witnessed	8121	44.7%	7963	5788	72.7%
			Bystander Witnessed	10,056	55.3%	9910	7979	80.5%
			Total	18,177	100.0%			
Urban or Rural	643	3.5	Rural Location	6030	34.4%	5963	5028	84.3%
			Urban Location	11,504	65.6%	11,278	8231	73.0%
			Total	17,534	100.0%			
Weekday or Weekend	0	0.0	Weekend	5553	30.5%	5453	4194	76.9%
			Weekday	12,624	69.5%	12,420	9573	77.1%
			Total	18,177	100.0%			
Transition Period (2015 & 2016)	0	0.0	Transition Period	14,089	77.5%	4010	3145	78.4%
			Other	4088	22.5%	13,863	10,622	76.6%
			Total	18,177	100.0%			
Post Transition (2017–2020)	0	0.0	Post Transition Period	9246	50.9%	8825	7274	82.4%
			Other	8931	49.1%	9048	6493	71.8%
			Total	18,177	100.0%			
COVID Period (2020)	0	0.0	COVID Period	15,857	87.2%	2296	1940	84.5%
			Other	2320	12.8%	15,577	11,827	75.9%
			Total	18,177	100.0%

**Table 1b:** *Bystander CPR in Ireland* in Ireland 2012–2020: Summary of continuous variables.

Variable	Available Cases	Missing		All		Bystander CPR		EMS initiated CPR	
		*n*	%	Mean	SD	Mean	SD	Mean	SD
				*Median*	*IQR*	*Median*	*IQR*	*Median*	*IQR*
Age (Years)	17,928	249	1.4	**63.2**	**19.7**	**63.2**	**19.6**	**63.5**	**20.1**
				67.0	52.0–78.0	67.0	52.0–78.0	68.0	52.0–79.0
Call Response (Mins)	17,755	422	2.3	**15.1**	**9.5**	**15.6**	**9.3**	**13.4**	**10.4**
				13.0	8.0–20.0	14.0	9.0–21.0	11.0	7.0–17.0

### Bystander CPR

**Fig. 2:**
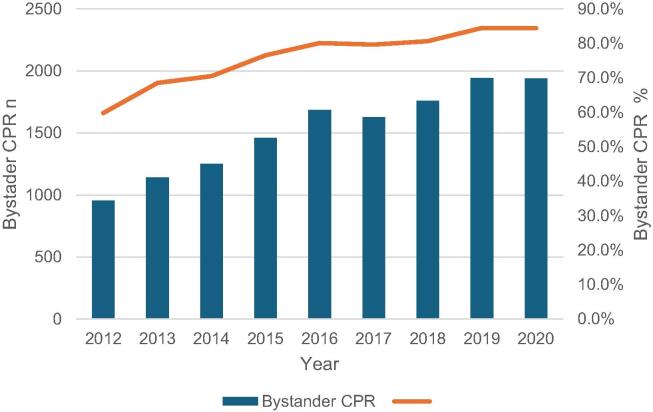
*Bystander CPR in Ireland* 2012–2020, absolute numbers and rates over time.

Fig. 2 illustrates the absolute numbers and rates of bystander CPR over the nine-year study time period. It demonstrates that the overall level of bystander CPR increased over time. Table 1a summarises bystander CPR proportions across predictor variable categories. Table 1b provides a summary of continuous predictor variables and illustrates the mean, standard deviation, median and interquartile range for all cases, cases where bystander CPR was performed and in cases where EMS initiated CPR. The overall rate of bystander CPR was 77.0%. The highest unadjusted rate of bystander CPR (84.5%) was seen during the COVID-19 period. The lowest rate was prior to the post transition period (71.8%). Median age (67.0 versus 68.0 years) was lower whereas median call response interval (14.0 versus 11.0 min) was higher in patients who had bystander CPR. supplementary Table 2 presents the results of univariate, unadjusted analysis. In this analysis the majority of predictors were associated with statistically significant p-values, with the exception of age, summer period and weekday.

Supplementary Table 3 and Table 2 present the results of the multivariable, adjusted analysis. During modelling quadratic terms for continuous variables were non-significant and thus dropped, this indicated that there was no substantial nonlinear relationship between the continuous predictors and the binary outcome on the log scale. Supplementary Table 3 summarises the results of the full and stepwise modelling. Model fit statistics (deviance and AIC) improved slightly from full to stepwise, to final model. In addition, the Hosmer-Lemeshow test revealed no model with inadequate fit.

**Table 2:** *Bystander CPR in Ireland* in Ireland 2012–2020: Multivariable analysis, Final Model with Interactions – Complete Cases and Multiple Imputation.

Predictor	Involved in Interactions	Complete Cases		Multiple Imputation	
		Odds Ratio (95% Confidence Interval)	*p*-value	Odds Ratio (95% Confidence Interval)	*p*-value
Presumed Cardiac Aetiology	*	3.00 (2.39, 3.75)	<0.001	3.00 (2.39, 3.75)	<0.001
Age (years)		0.992 (0.989, 0.994)	< 0.001	0.992 (0.990, 0.994)	< 0.001
Call Response Interval (minutes)		1.017 (1.012, 1.022)	< 0.001	1.017 (1.012, 1.022)	< 0.001
Home Location	*	1.18 (0.96, 1.45)	0.117	1.18 (0.96, 1.45)	0.117
Year		1.23 (1.16, 1.29)	<0.001	1.23 (1.16, 1.29)	< 0.001
Evening	*	1.39 (1.20, 1.61)	<0.001	1.39 (1.18, 1.56)	<0.001
Morning	*	1.36 (1.18, 1.56)	<0.001	1.33 (1.03, 1.72)	<0.001
Bystander Witnessed	*	1.33 (1.03, 1.72)	0.031	1.33 (1.03, 1.72)	0.031
Urban Location		0.52 (0.47, 0.57)	<0.001	0.52 (0.47, 0.57)	<0.001
Transition Period (2015 & 2016)		1.18 (0.99, 1.39)	0.062	1.18 (0.99, 1.39)	0.062
Post-transition period (2017–2020)		0.92 (0.70, 1.22)	0.582	0.92 (0.70, 1.22)	0.582
Covid Period (2020)		0.77 (0.65, 0.92)	0.004	0.77 (0.65, 0.92)	0.004
Interacting Variables					
Presumed Cardiac Aetiology * Home Location		0.32 (0.25, 0.40)	<0.001	0.32 (0.25, 0.40)	<0.001
Presumed Cardiac Aetiology * Bystander Witnessed		1.38 (1.09, 1.74)	0.007	1.38 (1.09, 1.74)	0.007
Evening * Bystander Witnessed		0.78 (0.64, 0.96)	0.02	0.78 (0.64, 0.96)	0.02
Morning * Bystander Witnessed		0.77 (0.63, 0.94)	0.009	0.77 (0.63, 0.94)	0.009

The pseudo *R*^2^ (Nagelkerke) of the final model was 11.2%. Tenfold cross validation was used to assess the predictive ability of the final model which was found to be 77.9%. The outputs of multiple imputation sensitivity analysis are included in Table 2 demonstrating complete case and multiple imputation analysis yielded similar results. Four of the ten predictor variables in the final model were involved in interactions (aetiology, incident location, time of day and who witnessed collapse).

Fig. 3a, Fig. 3b together provide an overall summary of the final model. Fig. 3a illustrates the (adjusted) odds ratios and associated 95% confidence intervals for the final model predictors that were not involved in interactions. In this adjusted model the COVID period was associated with reduced odds of bystander CPR (OR 0.77, 95%CI 0.65, 0.92), as were increasing age (OR 0.992, 95% CI 0.989, 0.994) and urban location (OR 0.52, 95% CI 0.47, 0.57). Call response interval (OR 1.017, 95% CI 1.012, 1.022), and advancing year (OR 1.23, 95% CI 1.16, 1.29) were associated with increased odds of bystander CPR. The transition period effect estimate was suggestive of increased odds of bystander CPR (OR 1.18), albeit with uncertainty in terms of confidence intervals (95% CI 0.99, 1.39). Fig. 3b summarises the final model predictor effects for those predictors that were involved in interactions, comparing effect estimates considering a base case scenario and other relevant interaction states. The baseline comparator status reflects all variables in the final model being at their baseline comparator state (presumed other (than cardiac) aetiology, other (than home) location, night-time, not witnessed, rural location, not the transition or post transition or COVID period. Fig. 3b illustrates that for the relevant population the odds of bystander CPR were increased when the event was of presumed cardiac aetiology (OR 3.00, 95% CI 2.39, 3.75). An interaction with bystander witnessed status further modified the effect estimate to a greater odds of bystander CPR (OR 4.14, 95% CI 3.32, 5.15), however a further interaction with home location modified the effect estimate downwards (OR 0.95, 95% CI 0.81, 1.12). In addition, for the relevant population morning and evening time were associated with increased odds of bystander CPR (OR 1.36, 95% CI 1.18, 1.56 & OR 1.39, 95% CI 1.20, 1.61). An interaction with bystander witnessed status modified these effect estimates somewhat downwards (OR 1.30, 95% CI 1.07, 1.58 & OR 1.27, 95% CI 1.04, 1.56).

**Fig. 3a:**
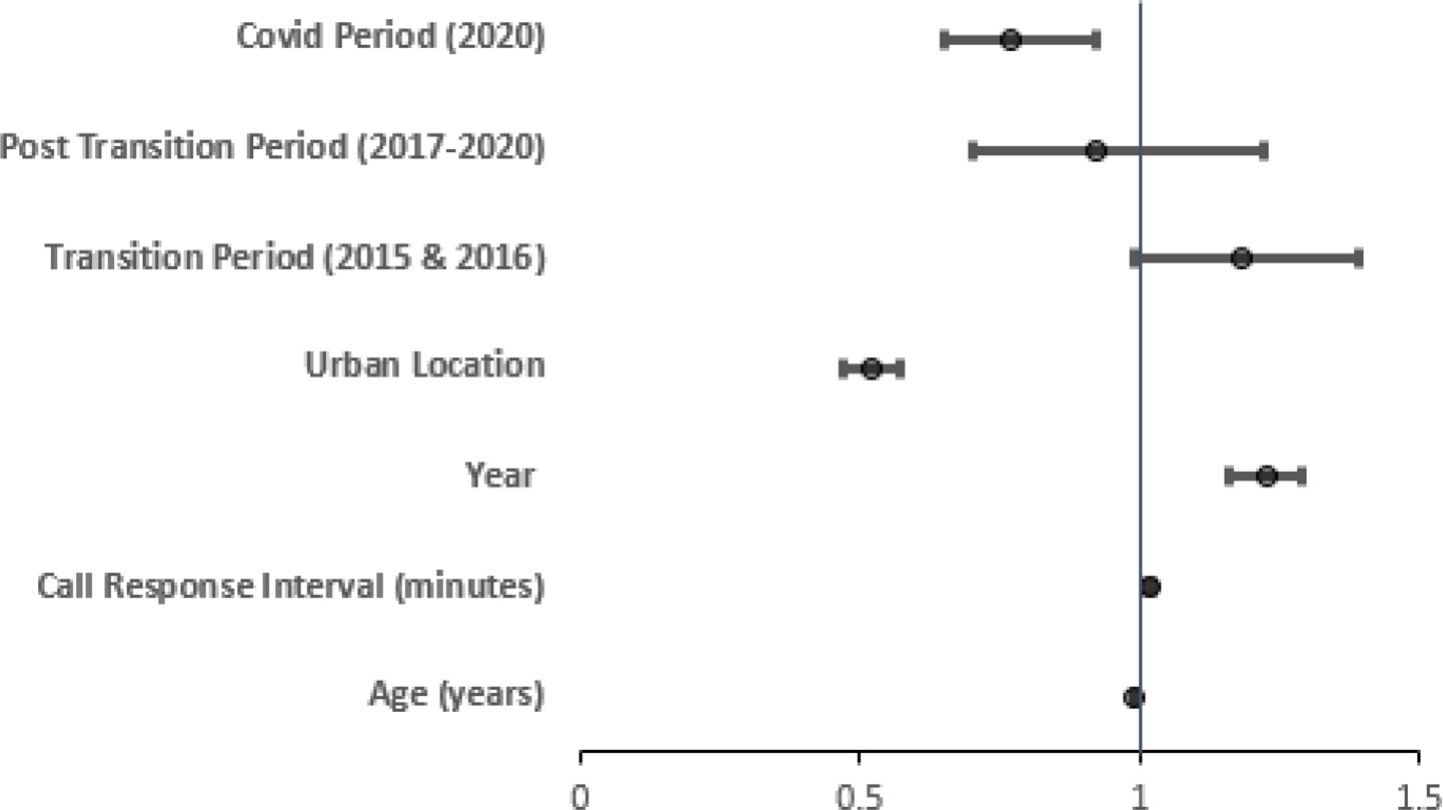
*Bystander CPR in Ireland* in Ireland 2012–2020: Multivariable analysis, Final model − Predictors without Interactions (odds ratios & 95% confidence intervals – interval scale).

**Fig. 3b:**
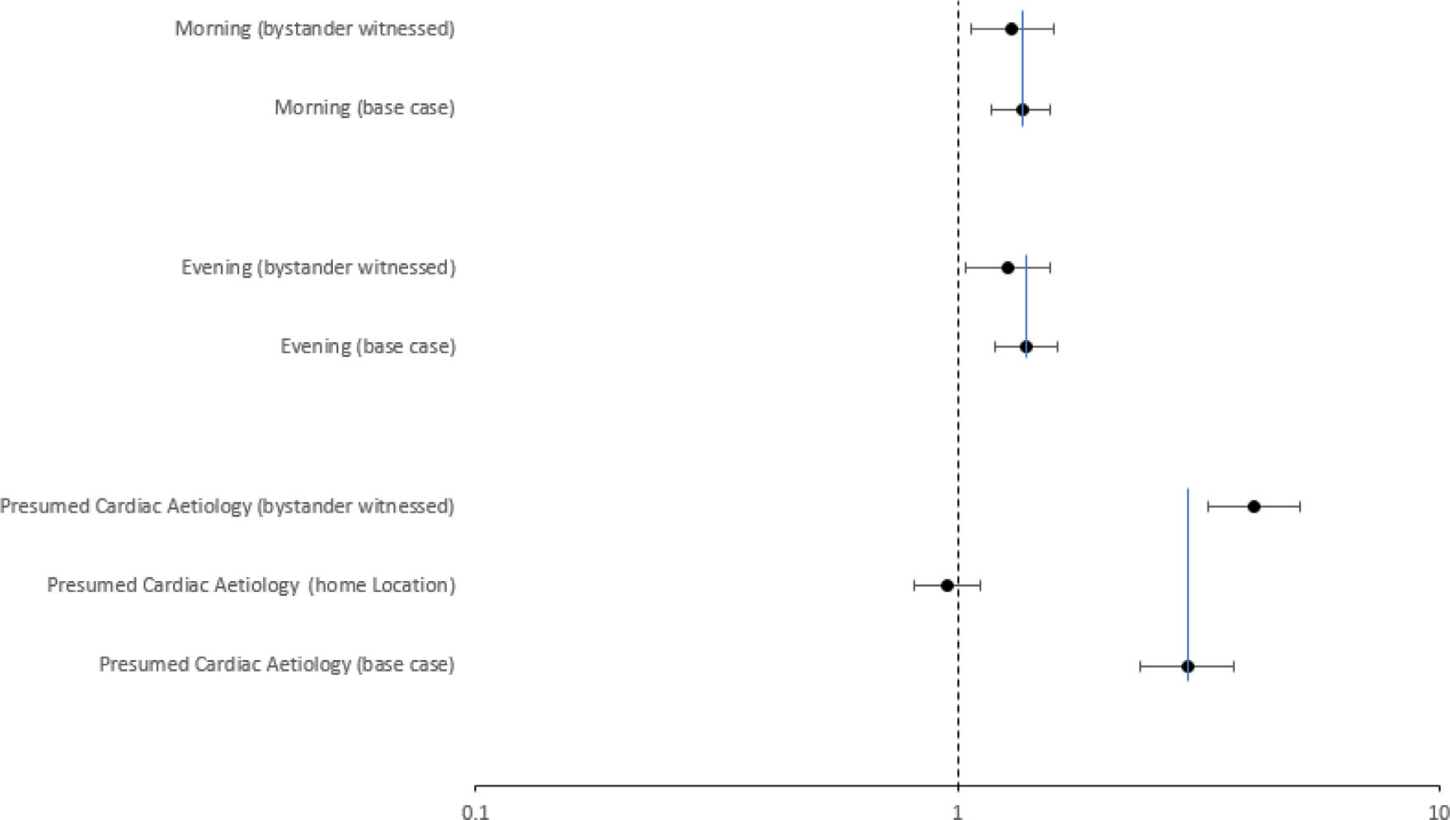
*Bystander CPR in Ireland* in Ireland 2012–2020: Multivariable analysis, Final model predictors with interactions (odds ratio & 95% confidence intervals – log scale).

## Discussion

Bystander CPR increased over the period 2012–2020 in Ireland and it is likely that pertinent health system developments contributed to this positive trend. In the final adjusted model, the period that marked the transition to a single NAS EMS control centre had an effect estimate that was suggestive of increased bystander CPR albeit with uncertainty in terms of confidence intervals. In the full model and stepwise models, which also passed goodness of fit assessments, the transition period was associated with increased odds of bystander CPR. In the final model the predictor year was also associated with increased odds of bystander CPR. Ultimately this suggests an incremental background year on year increase in bystander CPR with a probable parallel increase in bystander CPR associated with the transition period. Notably, the centralisation of EMS control in 2015 and 2016 was itself a component of a wider quality improvement programme launched in 2012 that continued throughout the period of the study. This programme incorporated community interaction and public education, call taking and dispatch, data management and audit.^8^ Some or all of these elements may have been drivers of increased bystander CPR over the study period. Other previous research has demonstrated that community-based interventions with and without health system interventions have been associated with improved rates of bystander CPR.^29^, ^30^ In the final adjusted model, the COVID period was associated with reduced odds of bystander CPR. In absolute terms though the rate of bystander CPR during the COVID period remained high (84.5%). However, it does appear that the sustained year on year increase in bystander CPR in Ireland was disrupted by COVID-19. Previous research by Tjelmeland et al that considered data from 26 international OHCA registries found an almost 5% annual increase in the incidence of bystander CPR from 2017 to 2020 and no change to the trend after lockdowns began.^31^ The research did however note variability at the level of individual registries with some noting decreased bystander CPR with COVID-19.^31^ Further in-depth research is warranted to explore bystander CPR in the specific context of the COVID 19-pandemic in Ireland.

In the final model increasing age in years was associated with decreased odds of bystander CPR, while increasing call response interval in minutes was associated with increased odds of bystander CPR. The change in odds for these predictors was small per unit year and minute, however given the range of ages and call response times present in the dataset still warrant ongoing consideration. Male sex was associated with increased odds of bystander CPR in univariate analysis, however this predictor was dropped from the multivariable models during variable selection. This suggest that sex on its own is not a principal driver of bystander CPR in Ireland. Previous research involving data from the Resuscitation Outcomes Consortium Registry demonstrated that males had an increased likelihood of receiving bystander CPR compared with females, but only in public locations.^32^ Further research based on data from the Pan-Asian Resuscitation Outcomes Study also noted that males were more likely to receive bystander CPR in public locations, however this research found that females were more likely to receive bystander CPR at home.^33^

Notably, urban location was associated with reduced odds of bystander CPR both in univariate analysis and in the final model which adjusted for call response interval amongst other variables. In absolute terms the rate of bystander CPR in urban areas was more than ten percentage points behind the rate in rural areas. This finding is significant, as a majority (65.6%) of OHCA events occurred in urban areas over the study period. Research from Denmark also found lower adjusted bystander CPR rates in urban areas.^34^ In the Irish context, bystander CPR in urban areas may represent a modifiable target to increase OHCA survival. Further research is now necessary to understand and address the urban–rural CPR disparity in Ireland. In all 86.0% of OHCA cases in this study were of presumed cardiac aetiology with 77.7% of patients in this category having bystander CPR versus 73.0% of those with another aetiology. In the final model presumed cardiac aetiology was involved in interactions with bystander witnessed status and with home location. Fig. 3a demonstrates that for the relevant population presumed cardiac aetiology was associated with increased odds of bystander CPR. The odds were further increased if the event was bystander witnessed but decreased substantially if the OHCA event occurred at home. These interactions suggest that key OHCA sub populations may be important to identify, study and better understand in terms of bystander CPR. Such populations include patients whose OHCA occurs at home and those patients who have an event that is not of a cardiac aetiology. Recent research using data from the Pan-Asian Resuscitation Outcomes Study analysed 13,631 cases of traumatic OHCA and found that bystander CPR was associated with increased odds of survival in a multilevel logistic model.^35^

This research has several limitations that must be considered. The research is observational in nature and cannot definitively establish whether the predictors studied directly influenced bystander CPR. In keeping with our protocol and following on from earlier research our modelling strategy undertook a statistically driven exploratory approach.^8^, ^24^ Follow up analysis focusing on key subgroups will be important and should be informed by clinically important elements of OHCA care. It is important to acknowledge the limitations of stepwise variable selection including the possibility that effect sizes may be inflated, and p-values too liberal. Thus, effect sizes and confidence intervals should be interpreted with a degree of caution and we have not made claims of effect based on statistical significance. The variable bystander CPR that was considered in this study was binary in nature. It captured only whether CPR was performed prior to EMS arrival. It did not capture who performed bystander CPR, the time at which CPR was initiated/performed or the quality of this CPR. Each of these unmeasured elements may have been significant in terms of outcomes. Recent research has highlighted large racial and ethnic differences in the incidence of bystander CPR in North America. The issue of race and ethnicity were not considered in this current study as OHCAR does not collect this data.^36^ This is a further limitation. A 2020 ILCOR scoping review considered the issue of willingness to perform bystander CPR.^37^ Although the study did not identify sufficient evidence to prompt a systematic review, it did highlight a significant knowledge gap in terms of factors that enhance the willingness of bystanders to perform CPR.^37^ Further research is needed to understand the issue of bystander CPR more fully, both in Ireland and internationally.

## Conclusions

Bystander CPR increased over the period 2012–2020 in Ireland. It is likely that the centralisation of EMS control contributed to this increase, while other health system developments including public education campaigns and the expansion of community first responders may also have contributed. Notably though, COVID-19 appeared to disrupt an otherwise positive trend. Urban location was associated with markedly decreased bystander CPR in both unadjusted and adjusted analysis. Bystander CPR in urban areas in Ireland should represent a public health target for intervention. More research is needed to understand and address variation in bystander CPR across different settings.

## CRediT authorship contribution statement

**Tomás Barry:** Writing – review & editing, Writing – original draft, Validation, Project administration, Methodology, Investigation, Funding acquisition, Formal analysis, Conceptualization. **Alice Kasemiire:** Writing – original draft, Formal analysis. **Martin Quinn:** Writing – review & editing, Writing – original draft, Data curation. **Conor Deasy:** Writing – review & editing, Writing – original draft, Methodology, Funding acquisition, Data curation. **Gerard Bury:** Writing – review & editing, Writing – original draft, Conceptualization. **Siobhan Masterson:** Writing – review & editing, Writing – original draft, Methodology, Investigation, Funding acquisition, Formal analysis, Data curation, Conceptualization. **Ricardo Segurado:** Writing – review & editing, Writing – original draft, Formal analysis. **Andrew W Murphy:** Writing – review & editing, Writing – original draft, Methodology, Data curation.

## Declaration of competing interest

The authors declare the following financial interests/personal relationships which may be considered as potential competing interests: ‘TB has research, clinical and educational roles in resuscitation care. He is a member of the Pre-Hospital Emergency Care Council (Ireland). All authors declare no conflict of interest.’.
