# Supplementary material for: Health systems developments and predictors of bystander CPR in Ireland
Source: Resusc Plus. 2024 May 31;19:100671. doi: 10.1016/j.resplu.2024.100671 (PMC11177081; doi:10.1016/j.resplu.2024.100671)
Supplement: Supplementary Data 1 [file mmc1.docx]

**Supplementary Figure 1:** Resuscitation for out-of- hospital cardiac arrest in Ireland 2012 -2020: Summary of health system temporal developments

(From Barry T, Kasemiire A, Quinn M et al. Outcomes of out-of-hospital cardiac arrest in Ireland 2012-2020: Protocol for an observational study [version 1; peer review: 1 approved]. HRB Open Res 2023, 6:17 (<https://doi.org/10.12688/hrbopenres.13699.1)>)

CFR: Community first responder, CPR: Cardio pulmonary resuscitation, GP: General Practitioner, NAS: National Ambulance Service, OHCA: Out-of-hospital cardiac arrest, PAD: Public access defibrillation,


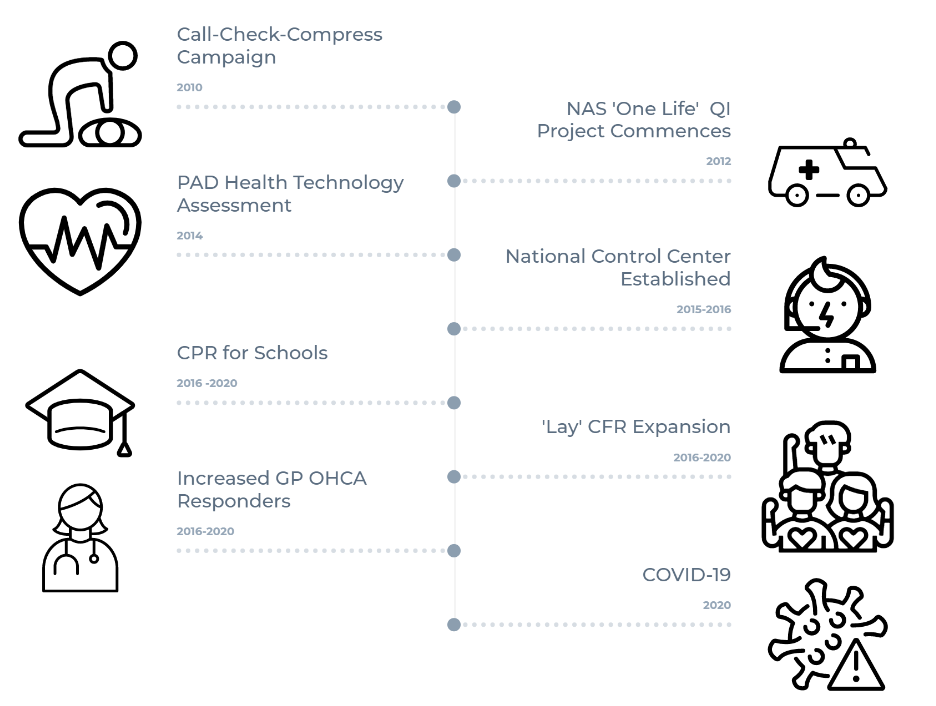


**Supplementary Table 1:** *Bystander CPR in Ireland* 2012–2020: Summary of variables for analysis

| Number | Variable | Categories | Collapsed Categories |
| --- | --- | --- | --- |
|  |  |  |  |
| 1 | Aetiology | Presumed Other | Trauma, Respiratory, Submersion, Non-cardiac, Other |
|  |  | Presumed Cardiac |  |
| 2 | Age | Age (years) |  |
| 3 | Sex | Female |  |
|  |  | Male |  |
| 4 | Call Response Interval | Call Response Interval (minutes) |  |
| 5 | Incident location | Other Location | Industrial, Public building, GP Surgery, Farm, Sport place, Residential institution, Street, Ambulance, Other |
|  |  | Home Location |  |
| 6 | Season | Winter | October-March |
|  |  | Summer | April -September |
| 7 | Year | Year (continuous variable 2012-2020) |  |
| 8 | Time of Day | Night (23:00–6:59 h) |  |
|  |  | Evening (15:00–22:59 h) |  |
|  |  | Morning (7:00–14:59 h) |  |
| 9 | Who Witnessed Collapse | Not Witnessed |  |
|  |  | Bystander Witnessed |  |
| 10 | Urban or Rural | Rural Location |  |
|  |  | Urban Location |  |
| 11 | Weekday or Weekend | Weekend |  |
|  |  | Weekday |  |
| 12 | Transition Period | Transition Period (2015 & 2016) |  |
|  |  | Not Transition Period |  |
| 13 | Post Transition Period | Post Transition Period ( 2017-2020) |  |
|  |  | Not Post Transition Period |  |
| 14 | Covid Period | Covid Period (2020) |  |
|  |  | Not Covid Period |  |

**Supplementary T****able 2:** *Bystander CPR in Ireland* in Ireland 2012 -2020: Univariate analysis

| Predictor | Univariate Model | |
| --- | --- | --- |
|  | Odds Ratio (95% Confidence Interval) | *p*-value |
| Presumed Cardiac Aetiology | 1.29 (1.17, 1.42) | <0.001 |
| Age (years) | 0.999 (0.997, 1.001) | 0.301 |
| Male | 1.15 (1.07, 1.24) | <0.001 |
| Call Response Interval (minutes) | 1.029 (1.025, 1.034) | <0.001 |
| Home Location | 0.54 (0.50, 0.59) | <0.001 |
| Summer (April-September) | 1.02 (0.95, 1.09) | 0.676 |
| Year | 1.17 (1.15, 1.19) | <0.001 |
| Evening | 1.26 (1.15, 1.39) | <0.001 |
| Morning | 1.22 (1.12, 1.34) | <0.001 |
| Bystander Witnessed | 1.55 (1.45, 1.67) | <0.001 |
| Urban Location | 0.50 (0.46, 0.55) | <0.001 |
| Weekday | 1.01 (0.94, 1.09) | 0.809 |
| Transition Period* (2015 & 2016) | 1.84 (1.67, 2.02) | <0.001 |
| Post Transition Period* (2017-2020) | 2.37 (2.19, 2.57) | <0.001 |
| COVID Period (2020) | 1.73 (1.53, 1.95) | <0.001 |

*Dummy variables adjusted for each other.

**Supplementary Table 3:** *Bystander CPR in Ireland* in Ireland 2012 -2020: Multivariable analysis, Full and Stepwise Models

| **Predictor** | **Full Model** | | **Stepwise Model** | |
| --- | --- | --- | --- | --- |
|  | Odds Ratio (95% Confidence Interval) | p-value | Odds Ratio (95% Confidence Interval) | p-value |
| Presumed Cardiac Aetiology | 1.57 (1.39, 1.77) | <0.001 | 1.56 (1.38, 1.76) | <0.001 |
| Age (years) | 0.991 (0.989, 0.994) | <0.001 | 0.991 (0.990, 0.994) | <0.001 |
| Male | 1.03 (0.95, 1.11) | 0.518 |  |  |
| Call Response Interval (minutes) | 1.017 (1.012, 1.021) | <0.001 | 1.017 (1.012, 1.021) | <0.001 |
| Home Location | 0.49 (0.45, 0.54) | <0.001 | 0.49 (0.44, 0.54) | <0.001 |
| Summer (April -September) | 1.04 (0.97, 1.13) | 0.261 |  |  |
| Year | 1.22 (1.16, 1.29) | <0.001 | 1.23 (1.16, 1.29) | <0.001 |
| Evening | 1.23 (1.11, 1.36) | <0.001 | 1.23 (1.11, 1.36) | <0.001 |
| Morning | 1.19 (1.08, 1.32 | <0.001 | 1.19 (1.08, 1.32 | <0.001 |
| Bystander Witnessed | 1.43 (1.32, 1.55) | <0.001 | 1.43 (1.33, 1.55) | <0.001 |
| Urban Location | 0.53 (0.48, 0.58) | <0.001 | 0.52 (0.48, 0.58) | <0.001 |
| Weekday | 0.99 (0.91, 1.07) | 0.850 |  |  |
| Transition Period (2015 & 2016) | 1.19 (1.00, 1.41) | 0.044 | 1.19 (1.00, 1.41) | 0.046 |
| Post Transition Period (2017-2020) | 0.94 (0.71, 1.24) | 0.645 | 0.93 (0.71, 1.24) | 0.633 |
| Covid Period (2020) | 0.78 (0.65, 0.93) | 0.005 | 0.78 (0.65, 0.93) | 0.005 |
